# Phytochemicals and antioxidative enzymes defence mechanism on occurrence of yellow vein mosaic disease of pumpkin (*Cucurbita moschata*)

**DOI:** 10.1007/s13205-012-0100-6

**Published:** 2012-11-07

**Authors:** Namrata Jaiswal, M. Singh, R. S. Dubey, V. Venkataramanappa, D. Datta

**Affiliations:** 1 Indian Institute of Vegetable Research, P/O Jakhini (Shahanshahpur), Varanasi, UP 221305 India; 2 Banaras Hindu University, Varanasi, UP India

**Keywords:** Catalase, Glutathione reductase, Begomovirus, Peroxidase, Superoxide dismutase

## Abstract

Pumpkin (*Cucurbita moschata*) samples showing yellow vein mosaic disease in Varanasi region were identified with begomovirus infection using PCR amplification. A sequencing analysis of the full genome revealed that it is a strain of *Tomato leaf curl Palampur virus* (GenBank ID. FJ931537). Phytochemical composition and antioxidative enzyme levels were compared in infected and healthy plants. The study revealed that the amount of total protein declined in the infected leaves but elevated up to 135 % in the fruits of infected plants, whereas vitamin C and antioxidants declined in infected leaves as well as fruits. There was substantial increase in total phenol content in leaves (72 %) and fruits (300 %) of infected plants. In infected samples, substantial increase in activities of superoxide dismutases (SOD), ascorbate peroxidase (APX), guaiacol peroxidase (GPX) and catalase (CAT) was observed as compared to the uninfected control plants. The native PAGE showed alterations in the intensities of isozyme bands in the infected plants. The APX, GPX, CAT, SOD and glutamate dehydrogenase (GDH) bands were intense in the infected plants, whereas the GR isozyme showed reduced intensity in diseased plants.

## Introduction

Pumpkin (*Cucurbita moschata*), an important *Cucurbitaceae* vegetable, is cultivated throughout tropical and subtropical regions of the world. Pumpkin yellow vein mosaic disease (PYVMD) is a major constraint for the cultivation of pumpkin in India (Jayashree et al. 1999; Muniyappa et al. 2003). Incidence of the disease can go up to 100 % under mono-cropping (Maruthi et al. 2003). Infected plants exhibit yellowing of veins in young leaves and intensive mosaic patches at later stages. The affected plants become stunted and exhibit premature flower drop. Although, attempts have been made to characterize the causal agent based on its biological characteristics, information on molecular biology of the causal organism is scant. Three species of geminiviruses causing PYVMD have been reported in India, namely *Squash leaf curl China virus*-India [India: Coimbatore: Pumpkin] (Muniyappa et al. 2003), *Tomato leaf curl New Delhi virus*-India [India: New Delhi: Pumpkin: 2006] (Maruthi et al. 2007), and *Tomato leaf curl Palampur virus*-India [India: Varanasi: Pumpkin: 2008] (ToLCPaV) (Jaiswal et al. 2011), all are bipartite. ToLCPaV is a recently identified unclassified begomovirus infecting tomato, cucumber (*Cucumis sativus* L.) and melon (*Cucumis melo*) (Kumar et al. 2008; Heydarnejad et al. 2009).

A variety of adverse environmental conditions or stresses like water deficit, pathogenesis, low and high temperature, water logging, etc. are known to cause oxidative damage to plants either directly or indirectly by triggering increased production of reactive oxygen species (ROS). The ROS produced by plants against invading pathogens have an important role in signal transduction and limiting pathogen progress (Peng and Kuc 1992; Kinily et al. 1993), but when produced in excess, they cause tissue necrosis (Elstner and Osswald 1994; Baker and Orlandi 1995). Therefore, an elevation of the antioxidative capacity of plants should increase their tolerance to necrosis induced by pathogens or abiotic stresses. Plant cells are protected against the oxidative damage caused by ROS through a complex antioxidant system, comprising antioxidants like ascorbic acid, glutathione and antioxidant enzymes like superoxide dismutases (SOD), catalases (CATs), peroxidase (PODs), and glutathione reductase (GR) which scavenge ROS within the tissues. Superoxide dismutase is the key antioxidative enzyme and catalyzes dismutation of superoxide free radical  into H_2_O_2_ and O_2_ (Treutter 2006). In turn, CAT and PODs like guaiacol peroxidase (GPX) and ascorbate peroxidase (APX) break down H_2_O_2_ in the living cells (Scandalios 1993).

Phenolic compounds are some among the most influential and widely distributed secondary products in plants. Phenolic compounds have long been correlated with the resistance of plants to infective agents (Scandalios 1993; Jabeen et al. 2009; Kumar et al. 2010). The ability of a plant to withstand pathogenic attack depends upon the coordination of different defence strategies.

The aim of the present study was (a) to identify the begomovirus strain causing yellow vein mosaic disease in pumpkin in Varanasi region (b) to examine the effect of viral infection on contents of phytochemicals in the tissues, and (c) to investigate the role of individual antioxidative enzymes in protection of pumpkin plants against oxidative damage caused by virus infection.

## Materials and methods

### Survey of PYVMD and virus transmission tests

Random surveys were conducted in different farmer’s fields around Varanasi, Utter Pradesh (India), to determine the incidence of PYVMD. During the survey, various symptoms like general yellowing of young leaves, curling, thickening of tender stems as well as erect and hard secondary branches, and severe to mild mosaic in the youngest leaves were predominantly observed. The percentage of disease was calculated as described by Maruthi et al. (2003). Further, the plants showing various symptoms were collected from fields and maintained in glasshouse conditions under continuous whitefly transmission. The culture of nonviruliferous whiteflies was originally collected and maintained on egg plants (*Solanum melongena* L.), which are confirmed by PCR for absence of begomovirus infection. This culture was used for whitefly transmission tests. Approximately, 100 adult whiteflies were collected and allowed to feed on infected pumpkin plants for 24 h for virus acquisition. Approximately 10 viruliferous whiteflies were then transferred to five healthy *C*. *moschata* 2-week-old seedlings for an inoculation access period (IAP) of 24 h. After inoculation, whiteflies were killed with sprayed insecticide (0.05 % imidacloprid). The inoculated seedlings were maintained in insect-proof cages and observed for symptom development for a period of 30 days, and symptoms were confirmed by PCR with coat protein specific primer pair. The inoculated seedlings were further used for the study of their phytochemical contents and antioxidative enzymes.

### DNA isolation

To determine the identity of begomovirus, total nucleic acids were extracted from symptomatic plants using the method described by Pich and Schubert (1993). The extracted DNA was diluted tenfold in sterile distilled deionised water before being subjected to PCR amplification then stored at −20 °C.

### PCR amplification

Total nucleic acid was isolated from symptomatic leaves of pumpkins and was checked for the presence of begomovirus infection by PCR with coat protein specific primer pair P1F: 5′-ATGGCGAAGCGACCAGC-3′ and P1R: 5′-TTAATTTGTTACGCAATCATA-3′. Further full-length DNA-A of the begomovirus(es) associated with PYVMD, was amplified by PCR using primers P2F: 5′–5′GTGGGGATCCATTATTGCACG-3′ and P2R: 5′CCGGATCCCACATG TTTGTAGA-3′ which were designed from previously characterized sequences of begomoviruses infecting pumpkins from NCBI database. DNA amplification was performed with 35 cycles of denaturation for 1 min at 94 °C, primer annealing for 45 s at 55–58 °C and primer extension for 1 min 30 s at 72 °C, with an initial denaturation at 94 °C for 3 min and a final extension for 15 min at 72 °C. The PCR reactions were carried out in a Gene Amp PCR system 9700 (PE Applied Biosystems, Foster City, CA) thermocycler. All amplifications were performed in volumes of 25 μL PCR mix containing 2 μL DNA template, 1.5 U *Pfu* polymerase, 25 mM MgCl_2_, 2 mM dNTPs and 25 pmol of each primer. PCR products were electrophoresed (1 h at 80 V) in 0.8 % agarose gels in Tris–borate-EDTA buffer, pH 8. Gels were stained with ethidium bromide (10 mg/mL) and viewed in a Gel documentation system (Alpha Innotech, USA).

### Cloning and sequencing

The PCR amplified fragment was purified using MiniElute gel Extraction kit (Qiagen). The purified product was cloned into Topo cloning vector (Invitrogen) and sequenced at Department of Biochemistry, Delhi University, South Campus, New Delhi. BLAST (Altschul et al. 1990) was used to compare the cloned nucleotide sequences with other begomovirus sequences available in the GenBank database. The sequences which showed more than 80 % similarity with present isolate were aligned using ClustalW program (Thompson et al. 1994), and phylogenetic analysis was carried out with MEGA 4.0 software (Tamura et al. 2011) using the default parameters of UPGMA. The bootstrapped consensus dendrogram was generated with 1000 replications.

### Phyotochemical analysis

Leaf and fruit samples were collected from the disease-free plants and artificially infected pumpkin (*Cucurbita moschata*) plants showing yellow vein mosaic symptoms. The samples were analyzed for total protein, vitamin C, total phenol and antioxidant activity. The vitamin C content of fresh samples was determined by the 2,6-dichlorophenol indophenol titration method (AOAC). For determination of total protein, 25 mg samples were digested with 5.0 ml of 2 M NaOH at 95–100 °C for 1 h. After centrifugation at 16,000×*g* for 10 min, the protein content in the supernatant was estimated by the method of Lowry et al. (1951) using bovine serum albumin (BSA, Sigma) as standard. Total phenols were extracted as described by Bray and Thorpe (1954), and calculated with standard curve prepared using catechol. Antioxidant activity was measured by coupled auto-oxidation of β-carotene and linoleic acid, and expressed as percentage inhibition relative to the control after 60 min of incubation (Emmons and Peterson 1999).

### Superoxide dismutase assay

The activity of SOD was assayed according to Misra and Fridovich (1972). Fresh tissues weighing 200 mg were homogenized in 5 ml of 100 mM K-phosphate buffer (pH 7.8) containing 0.1 mM EDTA, 0.1 % (v/v) Triton X-100 and 2 % (w/v) polyvinyl pyrrolidone (PVP). The extract was filtered through muslin cloth and centrifuged at 22,000x*g* for 10 min at 4 °C. Supernatant was dialyzed in cellophane membrane tubings against the cold extraction buffer for 4 h with 3–4 changes of the buffer and then used for the assay. The assay mixture in a total volume of 3 ml contained 50 mM sodium carbonate/bicarbonate buffer (pH 9.8), 0.1 mM EDTA, 0.6 mM epinephrine and enzyme. Epinephrine was the last component to be added. The adrenochrome formation in the next 4 min was recorded at 475 nm (extinction coefficient of 10.3 mM^−1 ^cm^−1^) using a mini 1240 UV–Vis spectrophotometer (Shimadzu). One unit of SOD activity is expressed as the amount of enzyme required to cause 50 % inhibition of epinephrine oxidation under the experimental conditions.

### Catalase assay

Fresh samples (200 mg) were homogenized in 5 ml of 50 mM Tris/NaOH buffer (pH 8.0) containing 0.5 mM EDTA, 2 % (w/v) PVP and 0.5 % (v/v) Triton X-100. The homogenate was centrifuged at 22,000x*g* for 10 min at 4 °C, and after dialysis the supernatant was used for enzyme assay according to Beers and Sizer (1952). The decomposition of H_2_O_2_ was followed at 240 nm (extinction coefficient of 0.036 mM^−1 ^cm^−1^) by observing decrease in absorbance. Enzyme specific activity is expressed as μmol of H_2_O_2_ oxidized min^−1^ (mg protein)^−1^.

### Guaiacol peroxidase assay

Fresh samples weighing 200 mg were homogenized in 5 ml of cold 50 mM Na-phosphate buffer (pH 7.0). The homogenates were centrifuged at 4 °C and the dialyzed enzyme extracts were used for the enzyme assay as described by Egley et al. (1983). Increase in absorbance was measured at 420 nm (extinction coefficient of 26.6 mM^−1 ^cm^−1^) at 30 s intervals up to 3 min. Enzyme specific activity is expressed as μmol of H_2_O_2_ reduced min^−1^ (mg protein)^−1^.

### Ascorbate peroxidase assay

Fresh samples weighing 200 mg were homogenized in 5 ml of 50 mM K-phosphate buffer (pH 7.8) containing 1 % PVP, 1 mM ascorbic acid and 1 mM PMSF as described by Moran et al. (1994). Ascorbate peroxidase was assayed according to Nakano and Asada (1981) and decrease in absorbance was recorded at 290 nm (extinction coefficient of 2.8 mM^−1 ^cm^−1^) at 30 s intervals up to 7 min. Correction was made for the low, non enzymic oxidation of ascorbic acid by H_2_O_2_. The specific activity of enzyme is expressed as μmol ascorbate oxidized min^−1^ (mg protein)^−1^.

### Glutathione reductase assay

Healthy and infected fresh samples weighing 200 mg were homogenized using chilled mortar and pestle in 5 ml of 50 mM Tris–HCl buffer (pH 7.6). The homogenate was centrifuged at 22,000×*g* for 30 min at 4 °C and the supernatant was dialyzed and Glutathione reductase was assayed according to Schaedle and Bassham (1977). The reaction was monitored by decrease in absorbance of NADPH at 340 nm (extinction coefficient of 6.22 mM^−1 ^cm^−1^). The specific activity of enzyme is expressed as μmol NADPH oxidized min^−1^ (mg protein)^−1^.

### Protein determination

In all the enzyme preparations, protein was determined by the method of Lowry et al. (1951) using bovine serum albumin (BSA, Sigma) as standard.

### Protein preparation for SDS-PAGE

After 30 days of inoculation, leaf samples were harvested. Soluble proteins were extracted by grinding 1 g freeze-dried sample with pestle and mortar in liquid nitrogen followed by extraction with 4 ml extraction buffer solution (250 mM sucrose, 25 mM Tris, pH 7.2) and centrifugation at 22,000x*g* for 20 min. SDS-polyacrylamide gel electrophoresis (PAGE) was performed following the method of Laemmli (1970).

### Isoenzyme profile of catalase

To determine the influence of viral infection on changes in isoforms and expression of catalase in growing pumpkin seedlings and fruits, catalase was extracted from leaf and fruit tissue and native PAGE was performed in vertical slab gels following the method of Davis (1964) at 4 °C. Tris–glycine (pH 8.3) was used as electrode buffer, 7.5 % running and 3.5 % stacking gels were used. Enzyme samples corresponding to 30 μg protein mixed with glycerol were layered on top of the stacking gel and electrophoretic run was completed using a current of 25 mA per slab. For detection of catalase isoforms, gels were soaked in 5 mM K-phosphate buffer (pH 7.0) and then transferred to a 5 mM H_2_O_2_ solution in the same buffer. After 10-min incubation, gels were rinsed with water and stained in a reaction mixture containing 2 % (w/v) potassium ferricyanide and 2 % (w/v) ferric chloride. The isozymes appeared as colourless bands on a deep blue background.

### Isoenzyme profile of ascorbate peroxidase (APX)

Native PAGE was performed by the method of Davis (1964) in 7.3 % polyacrylamide gels. To visualize enzyme isoforms, gels were incubated at room temperature for 15 min in 0.1 M Na-phosphate buffer (pH 6.4) containing 4 mM ascorbate and 4 mM H_2_O_2_. The gels were washed with water and then stained for 10 min with 0.1 % ferricyanide and 0.1 % ferrichloride (w/v) in 0.125 N HCl (Gara et al. 1993). Isoforms of APX appeared as colourless bands on a blue background.

### Isoenzyme profile of guaiacol peroxidase (GPX)

To determine the influence of viral infection in situ on changes in isoforms of GPX in growing pumpkin seedlings and fruits, enzyme samples corresponding to 40 μg protein were electrophoresed in 7.0 % running gels. For detection of GPX isoforms, gels were incubated at room temperature for 15 min in 10 mM K-phosphate buffer (pH 6.0) containing 20 mM guaiacol and 0.01 % H_2_O_2_. GPX isoforms were observed as dark brown bands on brown background.

### Isoenzyme profile of superoxide dismutase (SOD)

Native PAGE was performed in 7.5 % polyacrylamide gels. To visualize enzyme isoforms, after electrophoretic run at 4 °C, gels were incubated at room temperature for 30 min in dark in a mixture containing 10 mg NBT, 75 mg Na_2_EDTA and 3 mg riboflavin dissolved in 100 ml Tris–HCl buffer pH 8.2. Enzyme isoforms were visualized by illuminating the gels for 15 min.

### Isoenzyme profile of glutathione reductase

Isozymes of GR were separated on 9 % polyacryamide gels at 100 V for 2 h at 4 °C. Gels were soaked in 50 mM Tris–HCl buffer (pH 7.5) containing 10 mg MTT, 10 mg 2,6-dichlorophenol indophenol, 3.4 nM GSSG and 0.4 mM NADPH.

### Isoenzyme profile of glutamate dehydrogenase

Fresh samples weighing 200 mg were homogenized in 5 ml of ice cold 100 mM Na-phosphate buffer (pH 7.5) containing 1 mM disodium EDTA, 1 mM DTT and 1 % PVP. The homogenates were centrifuged and dialyzed enzyme extracts were used for isoenzyme profiling in 8.0 % polyacrylamide gel. After electrophoresis, gels were incubated in a mixture containing 20 mg NADP^+^, 30 mg nitroblue tetrazolium, 2 mg phenazine methosulphate, 25 ml of 0.5 M Na-phosphate buffer (pH 8.0), 5 ml of 1 M sodium glutamate (pH 7.0) and 70 ml water, for 2 h to visualize the bands.

## Results

### Survey and disease incidence

**Fig. 1:**
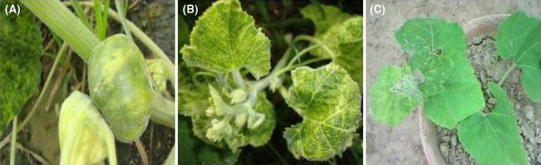
Leaves and fruit of pumpkin plants affected by *Tomato leaf curl Palampur virus* showing typical yellow mosaic symptoms (**a**, **b**) and test pumpkin plant (PKB-6) showing symptoms by whitefly (*Bemisia tabaci*) transmission (**c**)

During roving surveys, pumpkin plants exhibited different kinds of symptoms such as severe yellow-vein mosaics accompanied by leaf curl and stunted growth (Fig. 1). Symptoms of the disease were similar to those reported earlier by Maruthi et al. (2007) in the case of a whitefly-transmitted geminivirus which infects pumpkins. The incidence of symptomatic plants varied between fields at different locations and it was ranged from 40 to 80 % in mono cropping system.

### Disease transmission

**Fig. 2:**
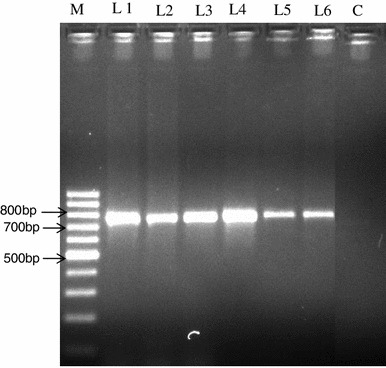
Amplification of coat protein (CP) gene in whitefly transmitted test pumpkin plants. *M* 100 bp DNA Ladder (Fermetas), *Lane 1* Positive control (virus infected plant) *Lane 2* to *Lane 6* PYVMV inoculated leaf samples, *C* negative control (buffer as a template)

The yellow vein mosaic disease samples collected from the field were transmitted through whiteflies on healthy seedlings of pumpkin plants by giving Acquisition Access Period and Inoculation Access Period of 24 h each. Three out of five inoculated plants showed severe yellowing symptoms after 20 days of inoculation, which was similar to that of naturally infected plants in the field. Based on positive whitefly transmission tests, the causal agent was suspected as a begomovirus. Therefore, PCR reactions were performed for inoculated plants using a primer designed from the coat protein (AV1) gene of the previously characterized sequence of begomoviruses infecting pumpkins from NCBI database and products of ~750 bp were obtained (Fig. 2).

### Detection of ToLCPMV and phylogenetic study

Pumpkin samples showing typical begomovirus symptoms of yellow vein mosaic disease were collected from several locations in Varanasi, and were tested positive for the presence of begomoviruses using coat protein specific primers with a PCR product of ~750 bp. The full genome of a representative sample was amplified using primers specific to DNA-A (P2F/P2R), then cloned and sequenced. The complete nucleotide sequence of DNA-A of begomovirus isolated from pumpkin was 2,756 nt long and the sequence is available in the NCBI Data base (accession No. FJ931537). The sequence contained typical features of old world bipartite begomoviruses, with two open reading frames (ORFs) [AV1 (CP), AV2] in virion-sense DNA-A and four ORFs [AC1 (Rep), AC2, AC3, AC4] in complementary-sense DNA-A, separated by an intergenic region (IR).

The full-length DNA-A sequence was compared with other begomoviruses available in the database, and it was found that the isolates showed maximum nucleotide identity of more than 99 % with *Tomato leaf curl Palampur virus* infecting tomatoes (AM884015). In general, for begomoviruses, the threshold cut-off value for distinguishing species from strains currently rests at 89 % (Fauquet et al. 2008) and the virus isolates displaying more than 90 % sequence identity should be considered as strains rather than different viruses (Padidam et al. 1995). These results indicate that the virus infecting pumpkins (*Cucurbita moschata*) is considered as a strain of *Tomato leaf curl Palampur virus* infecting tomato plants in India (Jaiswal et al. 2011).

### Effect of ToLCPMV on phytochemical contents

**Fig. 3:**
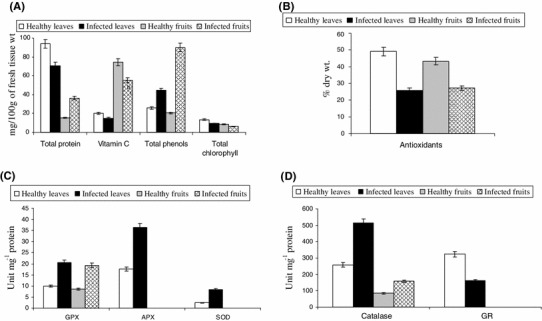
Effect of virus on value of **a** total protein, vitamin C, total phenol, **b** antioxidants, **c** activity of Superoxide dismutase (SOD), Ascorbate peroxidase (APX) and Guaiacol peroxidase (GPX) and **d** activity of Catalase (CAT) and Glutathione reductase (GR), in leaves and fruits. Values are mean ± SD based on three replicates, *bars* are significantly different at *P* ≤ 0.05

Phytochemical analyses of healthy and virus-infected leaves and fruits revealed that the total protein content declined in infected leaves but elevated up to 135 % in infected fruits (Fig. 3a, b), whereas ToLCPMV infection triggered 25 % reduction in vitamin C in the fruits. The antioxidants declined by 47 and 36 % in the infected leaves and fruits, respectively. There was a reduction of photosynthetic pigments (total chlorophyll, Chlorophyll a and b) in severely infected leaves. Total phenol was significantly higher in the infected leaves (73 % increases over the uninfected healthy leaves) and infected fruits (more than 300 % increase over the uninfected healthy fruits) (Fig. 3a, b).

### Effect of ToLCPMV on activities of antioxidative enzymes

The activities of antioxidative enzymes and their isoforms were analysed in non-inoculated healthy plants and infected plants 1 month after viral inoculation. In the infected leaf samples there was a substantial increase in the activities of SOD, APX, GPX and CAT as compared to the leaves of uninfected control seedlings. About 273 % increase in SOD and up to 100 % increase in APX, GPX and CAT were documented in the infected leaves, whereas about 49 % decline in GR activity was observed in the infected leaves of pumpkin seedlings (Fig. 3c, d).

**Fig. 4:**
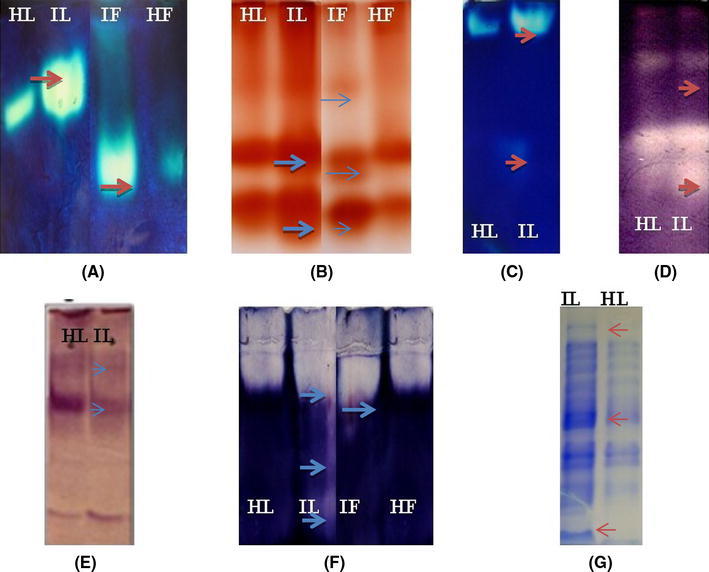
Isoenzyme profile during viral infection of **a** catalase, **b** guaiacol peroxidase, **c** ascorbate peroxidase, **d** superoxide dismutase, **e** glutathione reductase, **f** glutamate dehydrogenase and **g** SDS-PAGE pattern of soluble protein extracted from stressed and control seedlings. *Arrows* indicate the increase and decrease in intensity after infection. *HL* healthy leaves, *HF* healthy fruits, *IL* infected leaves, *IF* infected fruits

With quantitative changes in the enzyme levels, alterations were also observed in intensities and number of isozyme bands with infection. In-gel assays indicated the variation in intensities of APX, GPX, CAT, SOD, GR and GDH during stress. The APX, GPX, CAT, SOD and GDH bands were intensified, whereas GR isozyme showed reduced intensity with stress (Fig. 4).

### SDS-PAGE of soluble protein

Total soluble protein profile of the infected and healthy plants was compared. The protein profile of infected seedlings was different from healthy seedlings (Fig. 4g). The banding patterns also reflected differences in soluble protein contents.

## Discussion

Presently begomoviruses are emerging as a major threat for cultivation of crop plants in tropical and subtropical regions of the world (Polston and Anderson 1997). Characterization of pumpkin-infecting begomoviruses from India indicates the existence of at least two major viruses namely, SLCCNV in the South India and ToLCNDV in North India (Singh et al. 2009; Maruthi et al. 2007) associated with yellow vein mosaic disease of pumpkins. In the present study, another new strain of begomoviruses namely *Tomato leaf curl Palampur virus* (ToLCPaV) associated with yellow vein mosaic disease of pumpkin was identified. ToLCPaV is a recently identified unclassified begomovirus infecting tomato, cucumber (*Cucumis sativus* L.) and melon (*Cucumis melo*) (Kumar et al. 2008; Heydarnejad et al. 2009). In recent years, it is becoming increasingly evident that several natural and induced defence mechanisms operate in host plants against different biotic factors. The mechanism of host plant resistance in response to biotic stress consists of a series of changes in biochemical events such as emergence of free radicals, damage of cellular biomolecules, and consequently affects immune functions (Haliwell 1995; Bendich 1996).

The total protein level declined in leaves but was elevated nearly 135 % in fruits due to virus infection. Similar observations were reported for begomovirus-infected bitter gourd, where the total level of proteins was increased from 49 to 66 % (Raj et al. 2005). Content of vitamin C declined by 25 % and antioxidants level declined by 35–47 % in infected leaves and fruits. Total phenol level was significantly higher in diseased fruits (339 %) and leaves (73 %) as compared to healthy ones. Increased activity of polyphenol oxidase and phenylalanine ammonia lyase has been reported in plants treated with various biotic and abiotic inducers of resistance (Kumar et al. 2010; Raj et al. 2005; Huang and Backhouse 2005). Phenols play an important role in host pathogen interaction, disease development and defence reaction of infected plants (Treutter 2006; Jabeen et al. 2009; Kumar et al. 2010; Raj et al. 2005; Huang and Backhouse 2005). Hence, the increased quantity of phenolics in the infected parts of pumpkins presumably appears to contribute towards the resistance against viral infection.

Antioxidative enzymes play a crucial role in detoxification of ROS and in maintaining adequate level of antioxidants in the cells. Accumulation of the ROS in cells caused by environmental stresses results in concerted increase in the activities of antioxidative enzymes SOD CAT, GPX, APX (Peng and Kuc 1992; Kinily et al. 1993; Elstner and Osswald 1994; Baker and Orlandi 1995). The induction of antioxidative enzymes, such as SODs, POXs and CATs is the most common mechanism for detoxifying ROS synthesized during stress response (Mittler 2002; Kumar et al. 2009). Very few reports are available for antioxidative enzymes activity in plants subjected to biotic stresses especially, viral infection.

Peroxidases are important pathogenesis-related proteins (PR-proteins). They have important role in plant defence mechanisms, due to their involvement in the removal of hydrogen peroxide from the cells. Therefore, timing and localization of increased GPX and APX activity and their involvement in cell wall lignification, clearly suggested that peroxidases are involved in formation of barrier substances confined to the site of pathogen penetration (Pomar et al. 2002; Almagro et al. 2009).

In our studies, a significant enhancement of SOD (273 %), CAT (98 %), GPX (106 %) and APX (104 %) activities was observed in the infected plant parts, accompanied with increased H_2_O_2_ formation during viral infection. SOD, CAT, GPX and APX were over-expressed due to viral infection indicating their role in detoxification of ROS (Mittler 2002; Kumar et al. 2009). Another antioxidant enzyme, GR, showed reduced activity (Figs. 3d, 4e). Our observations are in agreement with the pattern reported during temperature and salt stress in French beans (Babu and Devaraj 2008) and in contrast to the patterns reported for low temperature stress in pea and maize, where increase in GR activity was observed under stress (Edwards et al. 1994; Prasad et al. 1995).

The data presented here, for the first time, confirms the presence of a new strain of begomovirus associated with yellow vein mosaic disease of pumpkins. The assessment of its impact on the levels of phytochemicals and activities of antioxidative enzymes of diseased and healthy plants, conducted in this study, is important for understanding the antioxidant defence mechanism in plants after viral infection. Furthermore, our results indicate that viral infection in pumpkin causes severe damage to the production and nutritional value of the plants, leading to deterioration of its quality, marked by lowering of vitamin C and antioxidant levels, and triggering increased activity of major antioxidative enzymes in the tissues.
